# The expression profiles and prognostic values of HSPs family members in Head and neck cancer

**DOI:** 10.1186/s12935-020-01296-7

**Published:** 2020-06-08

**Authors:** Guorun Fan, Yaqin Tu, Nan Wu, Hongjun Xiao

**Affiliations:** 1 Department of Otorhinolaryngology, Union Hospital, Tongji Medical College, Huazhong University of Science and Technology, Wuhan, 430022 China; 2 Department of Otolaryngology, Head and Neck Surgery, Stanford University School of Medicine, Stanford, CA USA

**Keywords:** Biomarkers, Prognosis, Expression, Heat shock proteins, HNSC

## Abstract

**Background:**

Head and neck squamous cell carcinoma (HNSC) ranks as the sixth most common malignancy. The identification of highly specific and sensitive prognostic markers and potential drug targets can contribute to enhanced patient prognosis and individualized treatments. Heat shock proteins (HSPs) act as molecular chaperones and play a crucial role in maintaining cell homeostasis. Recently, research has indicated that HSPs also act as “evil chaperones” in cancer development.

**Methods:**

In this study, we assessed the expression of HSPs in HNSC patients using the ONCOMINE, GEPIA, and UALCAN databases. Mutations of HSP genes were also analysed using the cBioPortal database. Additionally, the expression levels of HSPs were verified using the Human Protein Altas (THPA) database.

**Results:**

We found that the expression levels of HSPH1, HSPD1, SERPINH1, HSPA4, and HSP90AA1 were significantly higher in tissues from HNSC patients compared with normal tissues. Moreover, HSPH1, HSPD1, SERPINH1, HSPA4 and HSP90AA1 expressions were linked to disease progression. Survival analysis with the GEPIA and OncoLnc databases indicated that upregulation of HSPH1, HSPD1, SERPINH1, HSPA4 and HSP90AA1 was related to poor overall survival (OS).

**Conclusion:**

This study suggests that the HSPH1, HSPD1, SERPINH1, HSPA4 and HSP90AA1 genes are potential clinical targets and prognostic biomarkers for patients with HNSC.

## Background

Head and neck squamous cell carcinoma (HNSC) commonly occurs in the oral cavity, larynx, and pharynx and ranks as the sixth most common malignancy. In 2018, there was an estimated 43,000 HNSC-associated deaths worldwide and 835,000 new cases [1, 2]. Unfortunately, diagnosis of HNSC is usually made at an advanced disease stage as the clinical symptoms of HNSC are not obvious during the early stage. As a result, the current 5-year survival rate is less than 65% [3]. It is generally understood that the accumulation of genetic mutations in epithelial cells plays a key causal role in the development and progression of HNSC [4]. Hence, the identification of highly specific and sensitive prognostic markers and potential drug targets can contribute to enhanced patient prognosis and individualised treatments.

Heat shock proteins (HSPs) are genetically highly conserved proteins that act as molecular chaperones and play a crucial role in maintaining cell homeostasis [5]. Aside from their cytoprotective effects, recent research has suggested that HSPs also act as “evil chaperones” in the development, progression, metastasis and drug resistance of cancers [6, 7]. Therefore, HSPs have recently been proposed as potential therapeutic targets for tumor therapy [8]. HSPH1 (also named HSP105), a member of the HSP70 superfamily, is a component of the β-catenin degradation complex. Previous studies have demonstrated that overexpression of HSPH1/HSP105 in various cancers is associated with increased levels of nuclear β-catenin protein and upregulation of Wnt target genes [9]. HSPD1 is a molecular chaperone primarily localised in the mitochondrial matrix. It has been described as a potential prognostic and diagnostic biomarker for cancer. Recent studies have demonstrated that HSPD1 not only regulates the stability of survivin protein, but also regulates the mRNA expression of survivin [10]. SERPINH1, also called heat shock protein 47 (HSP47), is a collagen specific molecular chaperone. Several studies have confirmed that SERPINH1 participates in numerous steps of collagen synthesis, blocking the aggregation of procollagen and inducing the hydroxylation of proline and lysine residues. Abnormal expression levels of SERPINH1 are frequently found in a variety of cancers, including cervical, lung and gastric cancers [11–13]. Heat shock protein A4 (HSPA4), a member of the HSP110 family, is widely expressed in a variety of organs and can be induced under different conditions, including carcinogenic stress [14–16]. Recent studies have indicated that knockdown of HSPA4 can significantly reduce the migration, invasion, and transformation activities of tumor cells [17]. Heat shock protein 90α (Hsp90α) is the major cytosolic chaperone in eukaryotes. It is involved in cell protection and intracellular signaling transduction, controls intracellular homeostasis and assemblies of endoplasmic reticulum-secreted peptides, and regulates the translocation of proteins across the membranes of organelles after translation. Upregulated expression of Hsp90α is observed in a variety of cancer tissues, including liver, breast, and pancreatic cancers [18–20]. However, there is limited understanding of the underlying mechanisms and the unique roles of these genes in HNSC.

Although some studies have reported dysregulated expression of HSPs in HNSC and have linked this to patients’ prognosis [21, 22], the overall HSP expression profiles and the prognostic relevance of these expression profiles remain unknown. In the current study, we assessed the expression levels and mutations of HSPs in HNSC patients. The aim was to assess the potential functions, patterns of expression, and prognostic relevance of these genes in HNSC. To achieve this goal, we analysed large datasets available in various public databases.

## Methods

### Ethics statement

Our study was approved by the Academic Committee of Huazhong University of Science and Technology, and conducted in accordance with the principles expressed in the Declaration of Helsinki. All datasets used in this study were extracted from online public databases or published literature, and thus, written informed consent was not required for the current data analysis.

### ONCOMINE analysis

The expression patterns of HSPs in different cancers can be assessed using the online ONCOMINE database (https://www.oncomine.org). ONCOMINE is an integrated data-mining platform that store previously published or open-access cancer microarray data. The results were filtered by selecting HNSC vs. normal tissue.

### GEPIA dataset

GEPIA is an online database that incorporates gene expression data from TCGA and GTEx, bringing together 9736 tumor samples and 8587 normal controls. It allows for the assessment of differential expression profiles, patient outcomes, and various other analyses [23]. Through these public bioinformatics platforms, we are able to analyse the expression profiles of HSP genes in HNSC.

### UALCAN dataset

The UALCAN database (http://ualcan.path.uab.edu/index.html) is a user-friendly interactive platform for facilitating tumor subgroup gene expression and survival analyses [24]. Using the UALCAN database, we can evaluate the expression levels of HSP genes in HNSC and normal tissues based on tumor stages in the Cancer Genome Atlas (TCGA) HNSC datasets.

### The Human Protein Atlas (THPA) database

The Human Protein Atlas (THPA) (https://www.proteinatlas.org) is a public database that can be used to validate the expression of target genes. It contains immunohistochemical expression data for near 20 common kinds of cancers. In this study, we compared the protein expression levels of different HSPs between normal and HNSC tissues using immunohistochemistry images.

### Prognostic analysis

Using the GEPIA (http://gepia.cancer-pku.cn) database and OncoLnc (http://www.oncolnc.org) database, we analyzed the relationship between HSP gene expressions and overall survival in HNSC patients using a cox *p* value threshold of < 0.05.

### TCGA data and cBioPortal

The Cancer Genome Atlas (TCGA) database contains sequencing and clinicopathologic data for 30 cancer types [25]. The Head and Neck Squamous Cell Carcinoma (TCGA, Provisional) was selected for further analysis of HSP genes using cBioPortal (https://www.cbioportal.org). Genomic profiles, including mutations and copy-number alterations (CNA) were calculated using the cBioPortal’s online tool.

### Protein–protein interaction (PPI) network construction

In order to better understand the molecular mechanisms of HSPs in tumorigenesis, the PPI network for HSP family genes was constructed using STRING database. We choose a minimum interaction score of 0.4 as a cut-off when visualizing this PPI network.

### Functional and pathway enrichment analysis

The online STRING (https://string-db.org/) tool provides investigators with systematic and comprehensive functional annotation tools to identify the biological meaning behind an extensive list of genes. In our study, Gene ontology (GO) analysis and Kyoto Encyclopedia of Genes and Genomes (KEGG) enrichment analyses were conducted for HSP family genes using STRING. The significance threshold was *p* < 0.05.

## Results

### The expression levels of HSPs in HNSC patients

**Fig. 1:**
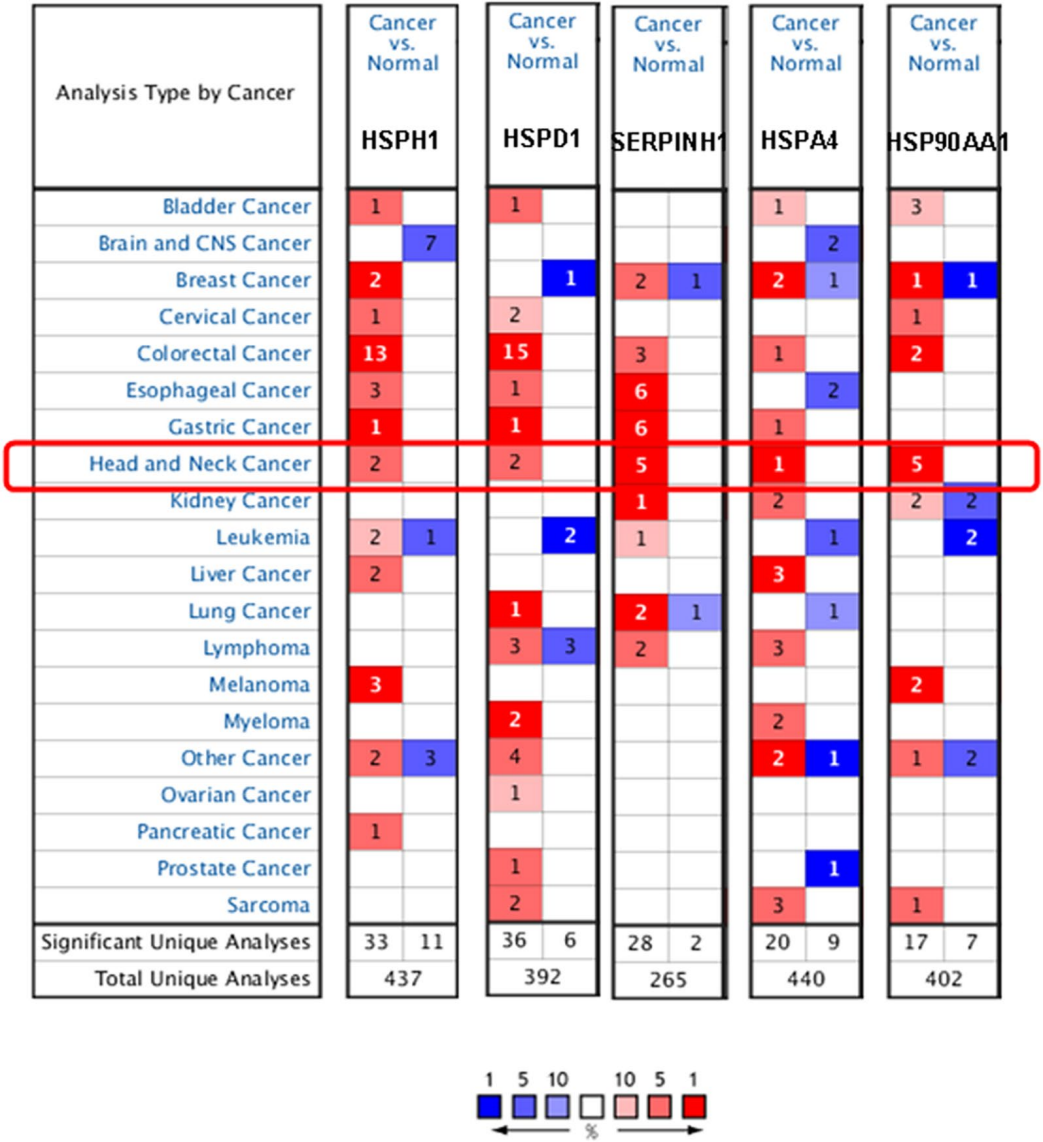
The expression levels of HSPs in different types of cancers (ONCOMINE). The expression levels of HSPH1, HSPD1, Serpinh1, HSPA4 and HSP90AA1 in different types of cancers. Red: over-expression; Blue: down-regulated expression

In order to explore the prognostic and potential therapeutic values of different HSP members in HNSC, we analyzed the mRNA and protein expressions of HSPs in HNSC patients using the ONCOMINE database. The expressions of five HSPs members (HSPH1, HSPD1, SERPINH1, HSPA4 and HSP90AA1) in more than 20 types of cancers were detected and compared with expressions in normal tissues using the ONCOMINE database. As were shown in Fig. 1, the expression levels of HSPs were significantly upregulated in HNSC patients. Interestingly, the expression levels of these five HSP genes were significantly upregulated in most tumors. In addition, we searched various HNSC datasets and found that these five genes showed significant upregulation across all datasets (Additional file 1: Fig. S1, Table 1). We further used the UALCAN.database to validate our findings. The results also showed that the expression levels of HSPH1, HSPD1, SERPINH1, HSPA4 and HSP90AA1 in HNSC were significantly increased (Additional file 2: Fig. S2).

**Table 1:** The significant changes of HSP associated genes expression levels between different types of Head and neck cancer and normal tissues (ONCOMINE database)

Gene	Types of head and neck cancer vs. normal	Fold change	p value	T test	Reference (PMID)
HSPH1	Tongue carcinoma vs. normal	2.902	5.56E−7	6.528	Pyeon, 17510386
	Tongue squamous cell carcinoma vs. normal	2.136	7.85E−5	4.433	Ye, 18254958
	Head and neck squamous cell carcinoma vs. normal	1.896	1.37E−5	5.107	Ginos, 14729608
	Tall cell variant thyroid gland papillary carcinoma vs. normal	1.178	3.22E−6	7.594	Giordano, 16609007
	Hypopharyngeal squamous cell carcinoma vs. normal	2.982	0.033	2.362	Schlingemann, 16205657
HSPD1	Salivary gland adenoid cystic carcinoma vs. normal	1.500	3.23E−4	4.102	FriersonHF, 12368205
	Head and neck squamous cell carcinoma vs. normal	2.282	4.67E−7	6.773	Ginos, 14729608
	Floor of the mouth carcinoma vs. normal	2.488	9.57E−7	7.144	Pyeon, 17510386
	Nasopharyngeal carcinoma vs. normal	1.812	3.60E−5	5.246	Sengupta, 16912175
	Tongue squamous cell carcinoma vs. normal	1.732	1.59E−5	4.785	Ye, 18254958
SERPINH1	Head and neck squamous cell carcinoma vs. normal	6.350	1.25E−6	12.064	Cromer, 14676830
	Tongue squamous cell carcinoma vs. normal	3.148	1.09E−10	7.757	Estilo,19138406
	Salivary gland adenoid cystic carcinoma vs. normal	308.933	3.74E−6	7.691	FriersonHF, 12368205
	Oral cavity squamous cell carcinoma vs. normal	2.072	2.44E−9	7.125	Peng,21853135
	Tongue squamous cell carcinoma vs. normal	2.022	2.05E−9	7.405	Talbot, 15833835
HSPA4	Thyroid gland oncocytic follicular carcinoma vs. normal	1.259	1.32E−4	6.863	Giordano, 16609007
	Oral cavity carcinoma vs. normal	2.732	4.49E−6	6.212	Pyeon, 17510386
	Tongue squamous cell carcinoma vs. normal	1.478	1.74E−6	5.146	Talbot, 15833835
	Follicular variant thyroid gland papillary carcinoma vs. normal	1.017	4.02E−4	3.464	TCGA, 2013
	Oral cavity squamous cell carcinoma epithelia vs. normal	1.199	0.002	3.391	Toruner, 15381369
HSP90AA1	Floor of the mouth carcinoma vs. normal	3.010	1.40E−8	8.051	Pyeon, 17510386
	Oral cavity carcinoma vs. normal	3.878	6.57E−9	8.959	Pyeon, 17510386
	Tongue carcinoma vs. normal	2.549	2.98E−7	6.220	Pyeon, 17510386
	Tonsillar carcinoma vs. normal	2.641	1.81E−6	6.050	Pyeon, 17510386
	Hypopharyngeal squamous cell carcinoma vs. normal	2.931	0.011	3.044	Schlingemann, 16205657

*TCGA* the Cancer Genome Atlas

### Correlations between the expression levels of HSPs and the clinicopathological parameters of HNSC patients

**Fig. 2:**
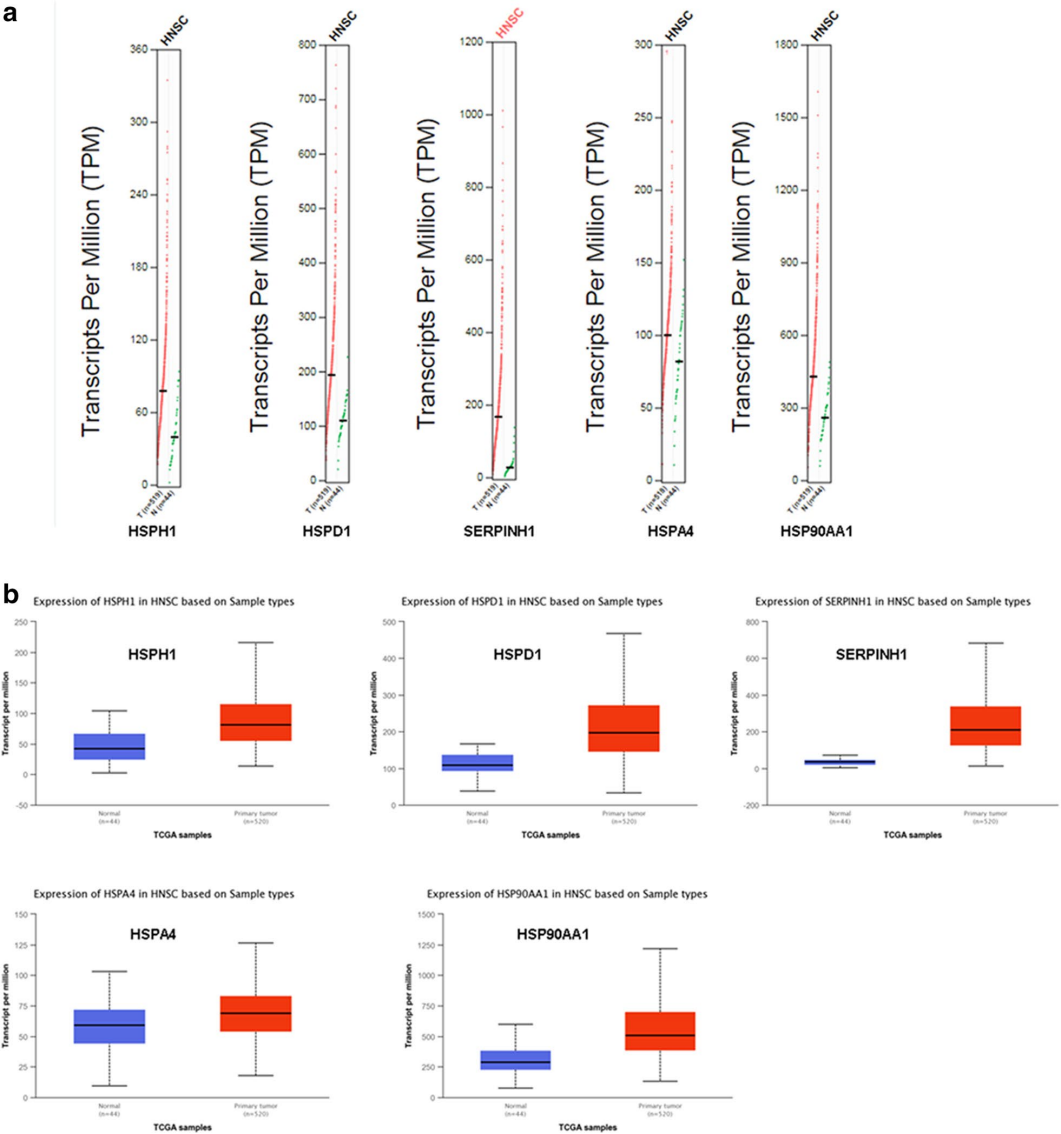
The expression profiles of HSPs in HNSC patients. The expression profile of HSPH1, HSPD1, Serpinh1, HSPA4 and HSP90AA1 in HNSC patients were analyzed by GEPIA (**a**) and UALCAN database (**b**); the p-value was set at 0.05

**Fig. 3:**
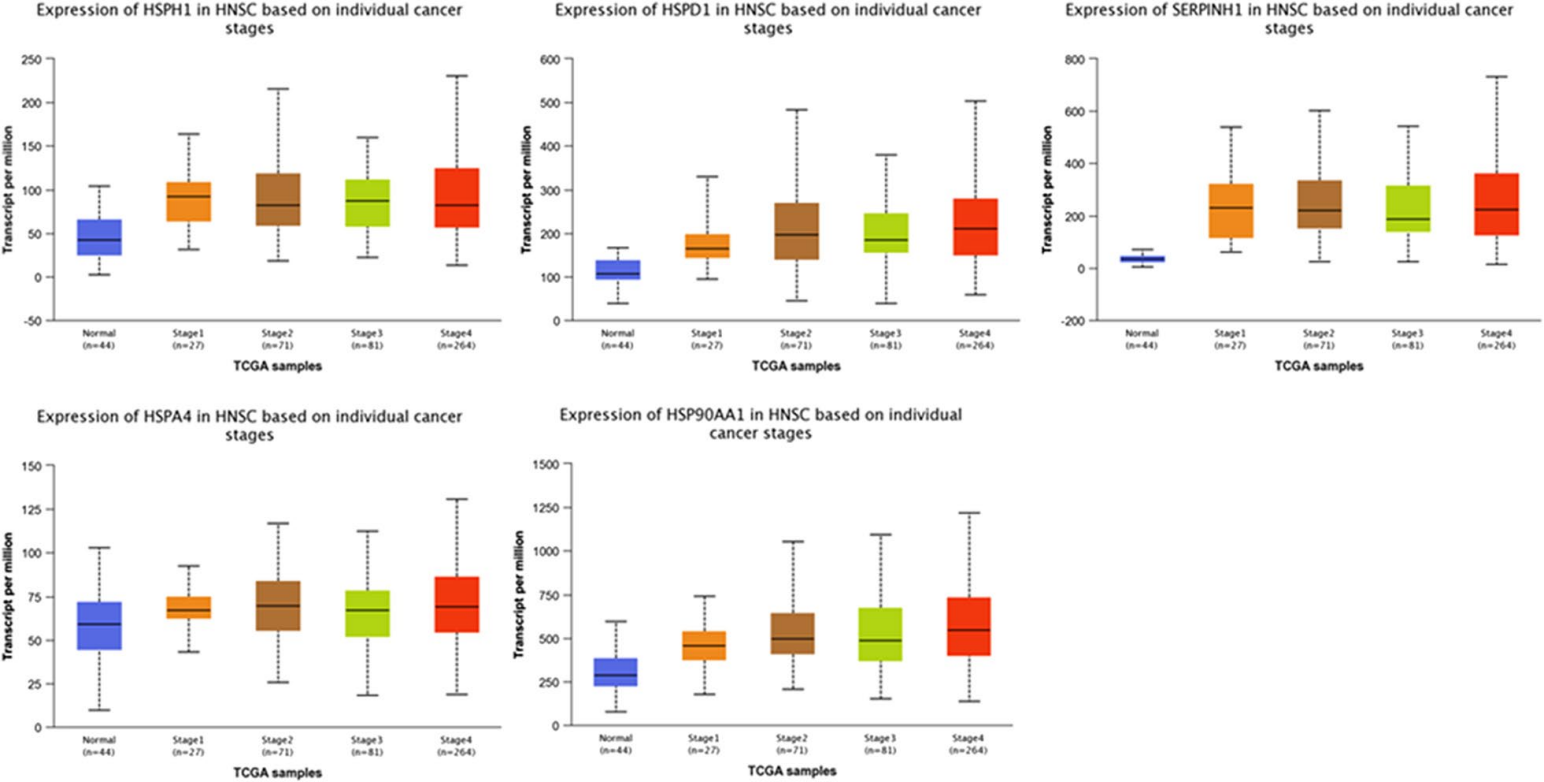
Correlations between expression levels of HSPs and tumor stage in HNSC patients. The relationships between expression levels of HSPH1, HSPD1, SERPINH1, HSPA4, and HSP90AA1 and tumor stages in HNSC patients were determined using the UALCAN database. The *p*-value was set at 0.05. The abscissa indicates the HNSC tumor stage and the ordinate indicates the expression levels of HSPs (gene expression ~ pathological stage)

**Fig. 4:**
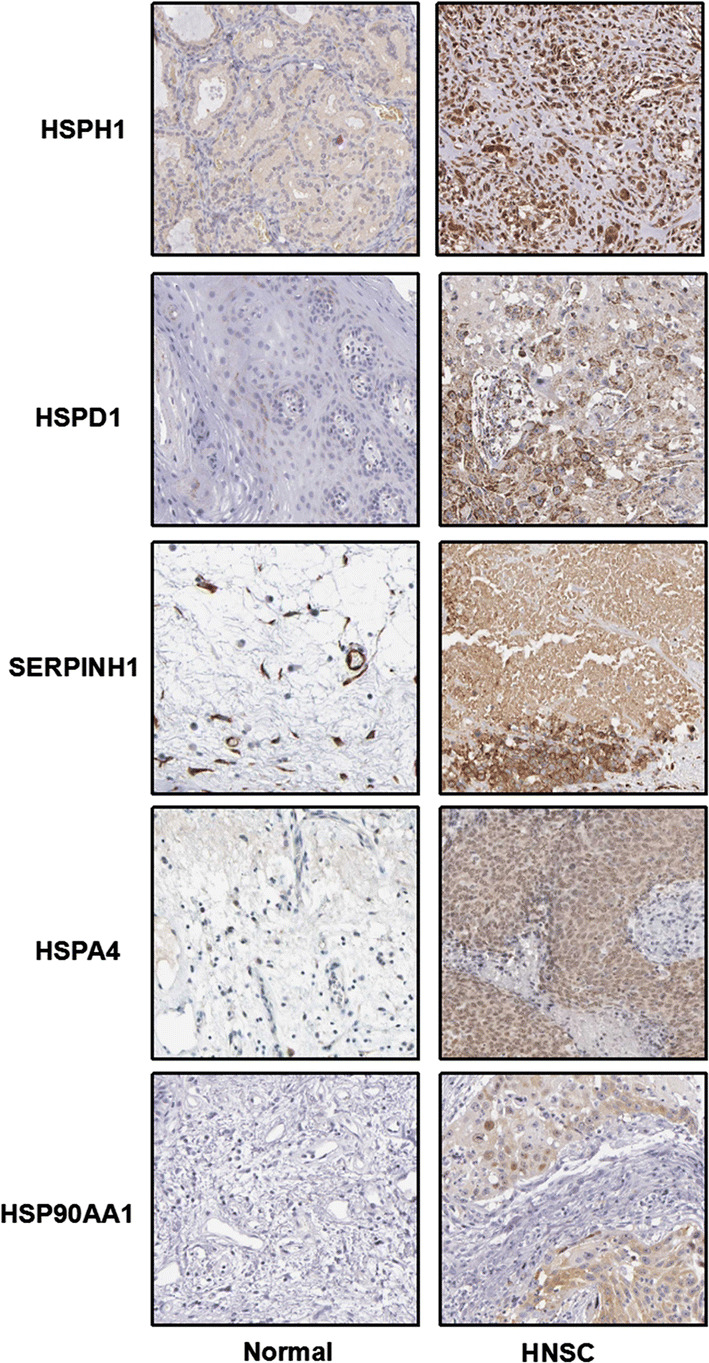
HSP genes were distinctively high expressed in HNSC tissues from Human Protein Atlas. HSP proteins were not expressed in normal head and neck tissues, whereas their high expressions were observed in HNSC tissues

GEPIA was used to compare HSPs between tumor and normal tissues. The findings revealed that the expression of all five genes was higher in HNSC samples than in normal control samples (Fig. 2a, b). We also analyzed HSPH1, HSPD1, SERPINH1, HSPA4, and HSP90AA1 expression as a function of the HNSC tumor stage. The results revealed a clear correlation between gene expression and tumor stage, with HNSC patients in more advanced stages tending to exhibit HSP expression levels (Fig. 3). After investigating the expression patterns of HSPH1, HSPD1, SERPINH1, HSPA4 and HSP90AA1 in HNSC, we examined HSP expression patterns in HNSC using THPA. The results confirmed that the protein levels of HSPH1, HSPD1, SERPINH1, HSPA4 and HSP90AA1 were elevated in HNSC samples relative to normal control samples (Fig. 4).

### Genetic alterations to HSP-associated genes and neighbor gene networks in HNSC patients

**Fig. 5:**
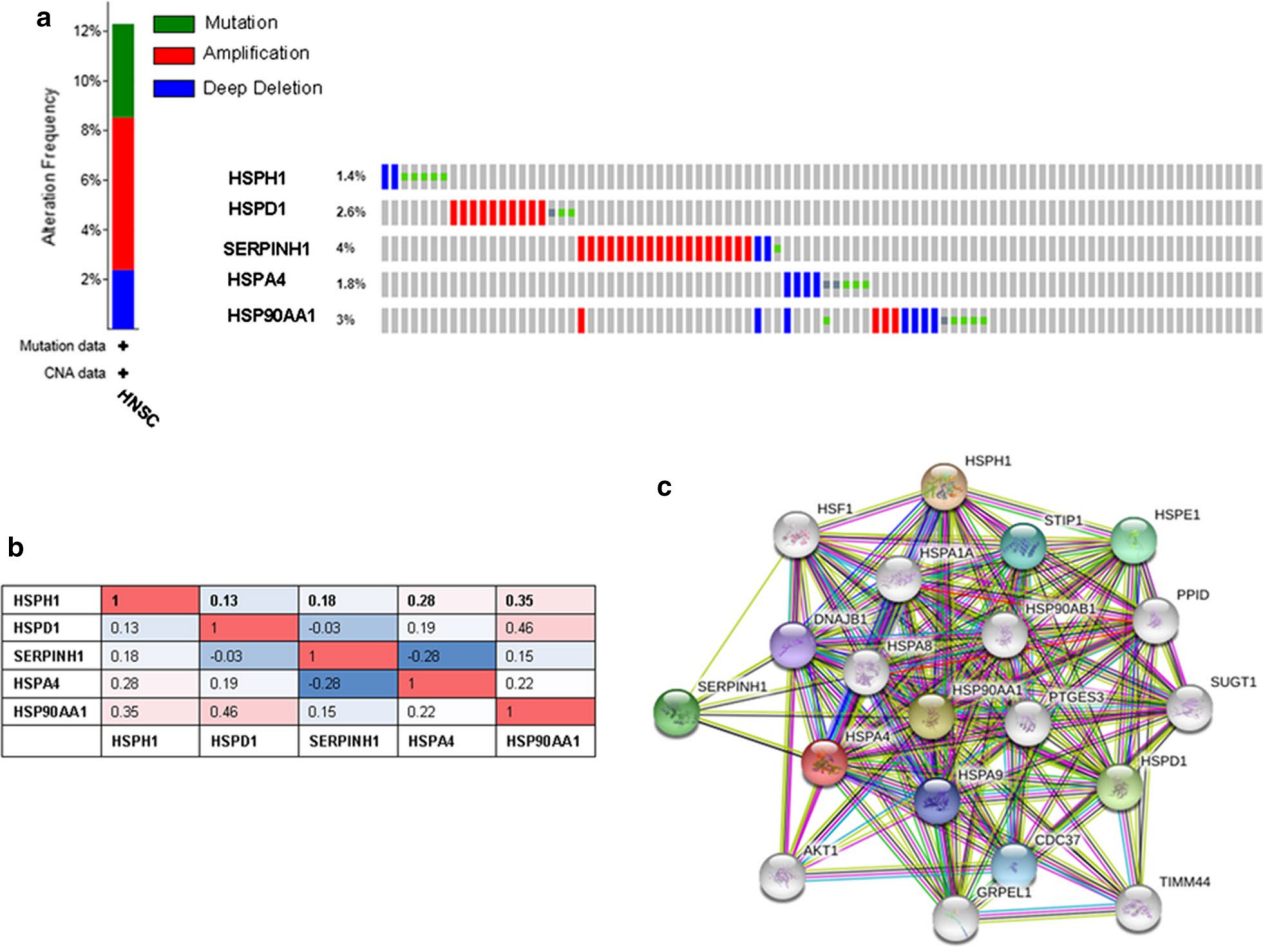
Expression of HSP genes and mutation analysis in HNSC. **a** Summary of alterations in HSPs. **b** Pearson correlation analysis of HSP family members. **c** Protein–protein interaction network of HSPs

The frequencies of mutations of the HSPH1, HSPD1, SERPINH1, HSPA4 and HSP90AA1 genes in HNSC were assessed using cBioPortal. In total, 504 patients from the Head and Neck Squamous Cell Carcinoma (TCGA, Provisional) were analyzed. According to this dataset, the percentages of genetic variations in HSPH1, HSPD1, SERPINH1, HSPA4 and HSP90AA1 genes among HNSC patients ranged from 1.4 to 4% for individual genes (HSPH1, 1.4%; HSPD1, 2.6%; SERPINH1, 4%; HSPA4, 1.8%; HSP90AA1, 3%). A total of 62 (12%) samples exhibited gene set/pathway alterations, with mutations in 2.82% of analyzed gene sets (Fig. 5a).The cBioPortal online tool also allows Pearson correlation analysis of HSP expression data (RNA Seq V2 RSEM) in HNSC (TCGA, Provisional). The results revealed a significant negative correlation between HSPD1 and SERPINH1 (Fig. 5b). Next, a PPI enrichment analysis, was then used to explore the relationships among these genes in HNSC. The PPI network was constructed by STRING. The results showed that several HSPs, including DNAJB1, HSPA1A, STIP1, HSPE1, HSPA8, HSPA9, HSF1, and HSP90AB1, were closely associated with HSPH1, HSPD1, SERPINH1, HSPA4 and HSP90AA1 (Fig. 5c).

### The prognostic values of HSPs in HNSC

**Fig. 6:**
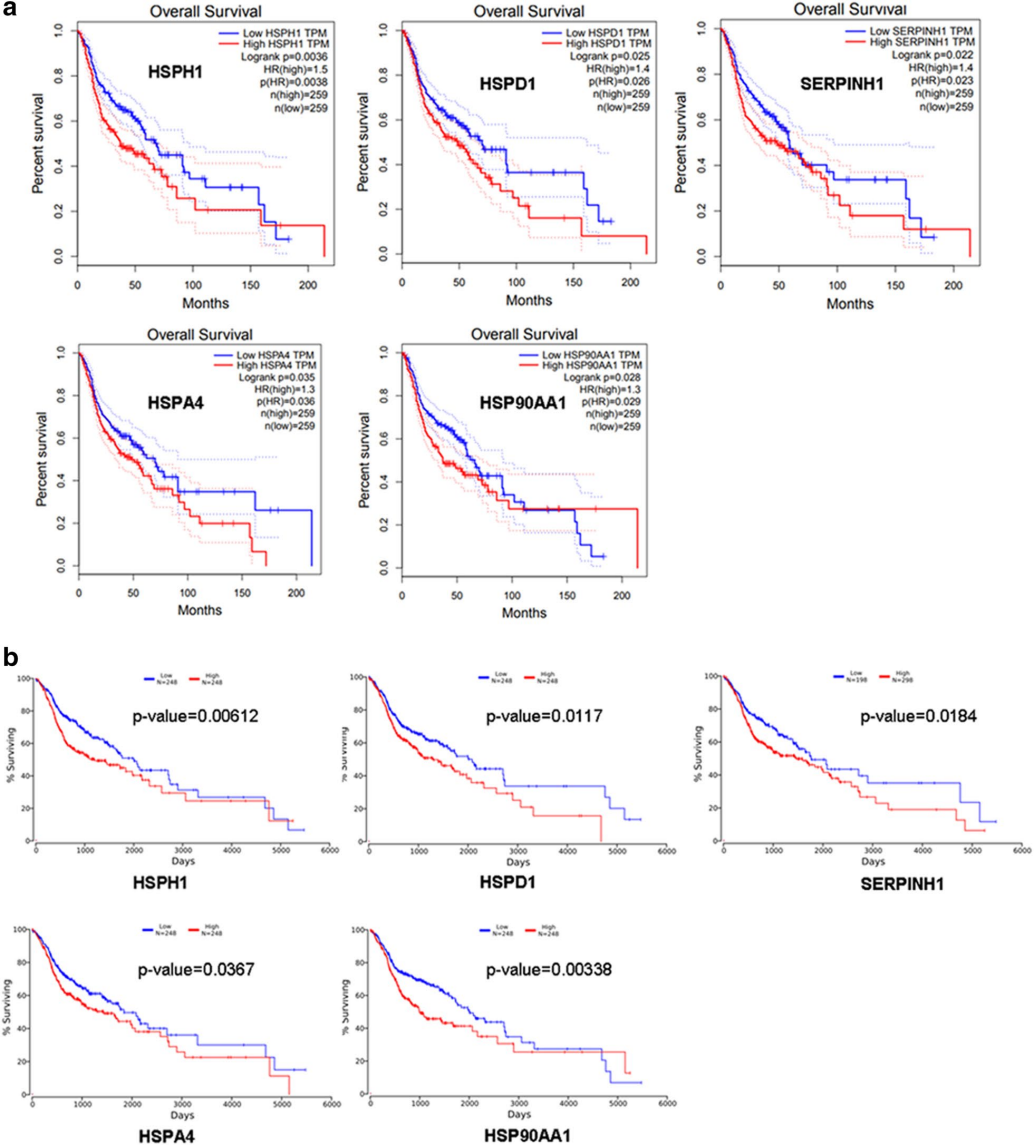
The prognostic values of HSP family members in HNSC patients. The overall survival curves comparing HNSC patients with high (red) and low (blue) HSPH1, HSPD1, SERPINH1, HSPA4, and HSP90AA1 expression levels were plotted using the GEPIA (**a**) and OncoLnc databases (**b**) at the threshold *p*-value of < 0.05

We evaluated the prognostic significance of HSPH1, HSPD1, SERPINH1, HSPA4 and HSP90AA1 in all HNSC patients using Kaplan–Meier plots and the OncoLnc database. The results revealed that increased expression levels of HSPH1, HSPD1, SERPINH1, HSPA4 and HSP90AA1 were strongly associated with poor overall survival (Fig. 6). Thus, the results suggest that highly expressed HSPs (HSPH1, HSPD1, SERPINH1, HSPA4 and HSP90AA1) are prognostic factors for HNSC. Since HSPs are reportedly associated with tumor immunity [26], we used the TIMER (https://cistrome.shinyapps.io/timer/) database to investigate the relationships between the HSP expression levels and the levels of immune infiltration in HNSC. Unfortunately, we did not find any significant correlations between HSP gene expressions and immune infiltration levels (Additional file 3: Fig. S3).

### Functional enrichment analysis of HSPs in HNSC patients

The functions of these five genes were next explored through GO and KEGG analyses. GO analyses allow assessment of the biological process, molecular function, and cellular component annotations of genes of interest. The results revealed that HSPH1, HSPD1, SERPINH1, HSPA4 and HSP90AA1 as well as their neighbor genes, are primarily enriched for regulation of protein ubiquitination, cellular protein metabolic and immune system process, chaperone-mediated autophagy, regulation of apoptotic process, positive regulation of DNA metabolic process, protein targeting to mitochondrion, regulation of cellular response to stress and heat, and protein folding (Additional file 4: Fig S4A). Enriched molecular functions included transcription regulation by Hsp70 protein binding, ATPase regulator activity, enzyme regulator activity, DNA polymerase binding, ubiquitin protein ligase binding, chaperone binding, ATP binding, protein binding, HSP binding, and unfolded protein binding (Additional file 4: Fig S4B). Cellular component annotations for these genes included mitochondrion, cytoplasmic vesicle lumen, intracellular organelle part, protein-containing complex, extracellular exosome, ficolin-1-rich granule lumen, cytosol, intracellular organelle lumen, cytoplasmic part, and chaperone complex (Additional file 4: Fig S4C). The KEGG pathways for these genes are shown in Table 2. Among these pathways, protein processing in the estrogen signaling pathway, endoplasmic reticulum, antigen processing and presentation, Prostate cancer, PI3K-Akt signaling pathway, NOD-like receptor signaling pathway, Epstein-Barr virus infection, MAPK signaling pathway, IL-17 signaling pathway, and Th17 cell differentiation were involved in tumor development and pathogenesis in HNSC (Additional file 5: Fig S5A and B).

**Table 2:** KEGG pathway analysis of HSP associated genes in HNSC

Pathway ID	Pathway name	Gene count	False discovery rate	Genes
hsa04141	Protein processing in endoplasmic reticulum	6	7.82E−07	DNAJB1, HSP90AA1, HSP90AB1, HSPA1A, HSPA8, HSPH1
hsa04612	Antigen processing and presentation	5	7.82E−07	HSP90AA1, HSP90AB1, HSPA1A, HSPA4, HSPA8
hsa04915	Estrogen signaling pathway	5	7.43E−06	AKT1, HSP90AA1, HSP90AB1, HSPA1A, HSPA8
hsa05215	Prostate cancer	3	0.0017	AKT1, HSP90AA1, HSP90AB1
hsa04151	PI3K-Akt signaling pathway	4	0.0033	AKT1, CDC37, HSP90AA1, HSP90AB1
hsa04621	NOD-like receptor signaling pathway	3	0.0045	HSP90AA1, HSP90AB1, SUGT1
hsa05169	Epstein-Barr virus infection	3	0.0061	AKT1, HSPA1A, HSPA8
hsa04010	MAPK signaling pathway	3	0.0173	AKT1, HSPA1A, HSPA8
hsa04657	IL-17 signaling pathway	2	0.0209	HSP90AA1, HSP90AB1
hsa04659	Th17 cell differentiation	2	0.0242	HSP90AA1, HSP90AB1

## Discussion

Dysregulation of HSPs is common in cancer development. Research suggests that HSPs are essential for tumor cell proliferation and differentiation. Although rarely used as diagnostic biomarkers for cancer, the expression levels of HSPs may predict the development of various cancers. For example, there is considerable evidence demonstrating that the over-expression of HSP27 may confer poor prognosis in gastric, prostate, and liver cancers [27–29]. Herein, we sought to investigate the expression pattern and prognostic values of different HSP members (HSPH1, HSPD1, SERPINH1, HSPA4 and HSP90AA1) in HNSC. These findings advance our current understanding of HNSC and may offer a means for improving treatment approaches and prognostic accuracy in HNSC patients.

The primary role of HSPs in tumorigenesis involves the stabilisation of functions of mutated or aberrantly-expressed tumor-related genes. Thus, high expression of HSPs is a hallmark of many cancers. In addition, HSPs are released from cancer cells, influencing their properties and functions through receptor-mediated signaling [30]. HSPH1 has been reported to be over-expressed in melanoma and colon cancer patients [31, 32]. The upregulation of HSPH1 is implicated in hyperactivation of the Wnt signalling pathway. The transcription levels of Wnt signalling target genes are significantly downregulated in cell line models with HSPH1 inhibition [9]. In our study, we confirmed that the expression of HSPH1 in HNSC tissue was significantly elevated compared with normal tissue. We also observed a significant correlation between the expression of HSPH1 and tumor stage in HNSC patients. High HSPH1 expression was associated with low overall survival in all HNSC patients.

HSPD1 is a molecular chaperone that is primarily localized in the mitochondrial matrix [33]. Recently, HSPD1 has been found in many extramitochondrial sites, including the extracellular surface, cell surface, intracellular vesicles, nucleus, extracellular fluid, and even the cytoplasm. HSPD1 has been recognized as a potential biomarker for tumor diagnosis and prognosis, especially in colorectal cancer. There is increasing evidence that HSPD1, especially intracellular HSPD1, is involved in the survival and metastasis of various tumors [34–36]. In our study, the ONCOMINE and TCGA datasets revealed that the expression of HSPD1 was higher in HNSC tissue than in normal tissues. High HSPD1 expression was significantly associated with poor overall survival among HNSC patients followed up for more than 6000 days.

SERPINH1 is a collagen-binding protein and has been identified to be a collagen-specific chaperon. It promotes the malignant behavior of cancer cells and predicts the metastatic activity of human cancer cells. SERPINH1 also plays an essential role in regulating the expression of extracellular matrix (ECM) proteins and fibronectin (FN); therefore, dysfunction of SERPINH1 stimulates abnormal expression of ECM proteins, which promotes epithelial-mesenehymaltransition (EMT) [37]. Our data confirmed that dysregulation of SERPINH1 is closely related to the development and progression of HNSC, and further affects the prognosis of HNSC.

HSPA4 is highly expressed in malignant tumor cells and is involved in tumor development and chemotherapy resistance, presumably due to its ability to inhibit tumor cell apoptosis [38]. It has been reported that overexpression of HSPA4 can inhibit cell apoptosis and prevent activation of the caspase signaling pathway, which leads to accumulation of misfolded proteins, ROS, and DNA damage. HSP70 not only affects the apoptotic pathway, but also the autophagic pathway. It prevents the formation of autophagosomes by activating the mTOR pathway. Consistent with these results, Leu et al. found that the inhibition of HSP70 resulted in significant increases in the expression of LC3-II and the number of autophagosomes [39]. In our study, it is clear that HSPA4 acts as an “evil chaperone” in HNSC. Its expression was significantly increased in HNSC patients and was associated with poor prognosis.

HSP90AA1 acts as a highly conserved chaperone protein and participates in tumor cell differentiation, proliferation, and angiogenesis. Recently, it has been regarded as a promising target for specific cancer therapy [40]. Hsp90AA1 is expressed in various cancers, including breast, colon, ovarian, lung and prostate cancers. This may be related to the involvement of HSP90AA1 in the regulation of apoptosis and signaling transduction triggered by growth factors, death receptors, and stress signals [41]. HSP90AA1 inhibits the initiation of apoptosis by blocking the binding of caspase 9 to apoptotic protein 1 activator. Additionally, it promotes the formation of tumor cells by stabilizing mutant p53 complexes, thereby inhibiting the apoptosis of tumor cells [38, 42]. In our systematic analyses of various databases, we found that HSP90AA1 was significantly upregulated in HNSC patients, and its expression level was significantly associated with the tumor stage in HNSC patients. As expected, high HSP90AA1 expression predicted poor overall survival among HNSC patients, implying an oncogenic role of HSP90AA1 in HNSC.

In this study, we also explored the genetic alterations and potential functions of HSP family members. The percentages of genetic mutations in HSPs in HNSC varied from 1.4% to 4% for individual genes. At the same time, we constructed a network for these five genes and their neighbor genes. Functional analysis demonstrated that these five genes were primarily enriched in tumor-related signaling pathways, indicating that HSPH1, HSPD1, SERPINH1, HSPA4, and HSP90AA1 play crucial cancer-promoting roles in the development of head and neck cancer.

## Conclusion

In summary, our results indicate that HSPH1, HSPD1, SERPINH1, HSPA4, and HSP90AA1 are significantly upregulated in HNSC patients and their upregulation is negatively correlated with HNSC tumor stage. Based on the above findings, it is expected that HSPH1, HSPD1, SERPINH1, HSPA4, and HSP90AA1 could act as potential prognostic biomarkers and therapeutic targets for HNSC. Our research contributes to a better understanding of the pathogenesis of HNSC and may assist in the development of more effective targeted drugs for head and neck cancer. However, further mechanistic studies are needed to validate our findings and to promote clinical application of HSPs as prognostic or therapeutic targets in HNSC.

## Supplementary information

**Additional file 1: Fig. S1.** The expression levels of HSPs in HNSC (ONCOIME). The expression levels of HSPH1, HSPD1, Serpinh1, HSPA4 and HSP90AA1 in several head and neck cancer studies. Red: over-expression. The significance threshold was *p* < 0.05.

**Additional file 2: Fig. S2.** Expressions of HSPs across TCGA cancers (with tumor and normal samples). The expression levels of HSPH1, HSPD1, Serpinh1, HSPA4 and HSP90AA1 in pan-cancer.

**Additional file 3: Fig. S3.** The relationships between the HSP expression levels and the levels of immune infiltration in HNSC.

**Additional file 4: Fig. S4.** Functional Enrichment Analysis of HSPs in patients with HNSC. GO enrichment analysis predicted the function of target genes from three aspects: biological processes (A), cellular components (B), and molecular functions (C).

**Additional file 5: Fig. S5.** p53 signal pathway and cell cycle pathway regulated by HSPs in HNSC. The MAPK signal pathway (A) and PI3K-Akt signaling pathway (B) regulated by HSPs in HNSC are shown.

## Data Availability

All data generated or analyzed during this study are included in this published article.

## Abbreviations

HNSC: Head and neck squamous cell carcinoma
HSPs: Heat shock proteins
THPA: The Human Protein Altas
OS: Overall survival
HSP47: Heat shock protein 47
Hsp90α: Heat shock protein 90α
ECM: Extracellular matrix
TCGA: The Cancer Genome Atlas
CAN: Copy-number alterations
GO: Gene ontology
KEGG: Kyoto Encyclopedia of Genes and Genomes
