# Supplementary figures and images for: The expression profiles and prognostic values of HSPs family members in Head and neck cancer
Source: Cancer Cell Int. 2020 Jun 8;20:220. doi: 10.1186/s12935-020-01296-7 (PMC7278206; doi:10.1186/s12935-020-01296-7)

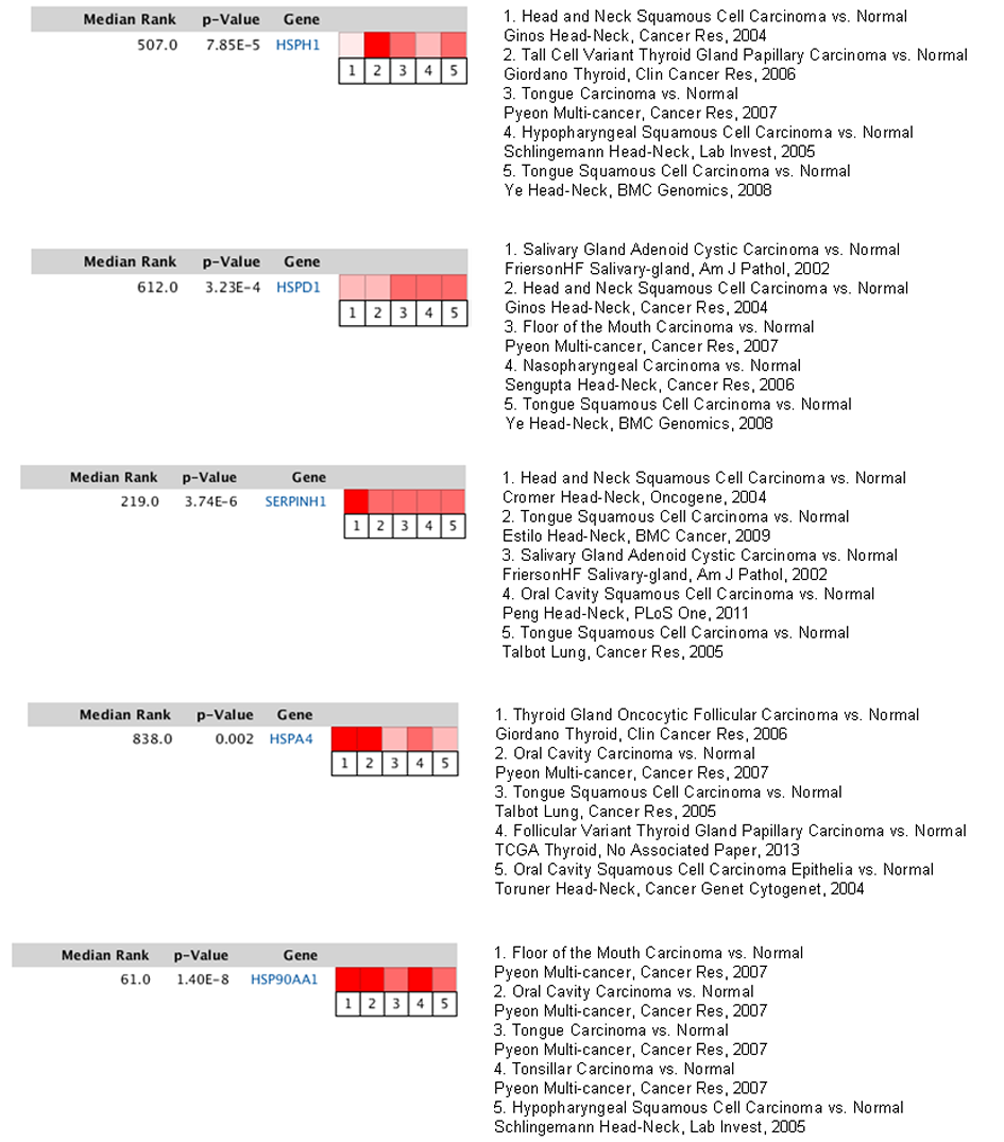

Supplement: Supplementary file 1 — Additional file 1: Fig. S1. The expression levels of HSPs in HNSC (ONCOIME). The expression levels of HSPH1, HSPD1, Serpinh1, HSPA4 and HSP90AA1 in several head and neck cancer studies. Red: over-expression. The significance threshold was p < 0.05. [file 12935_2020_1296_MOESM1_ESM.tif]

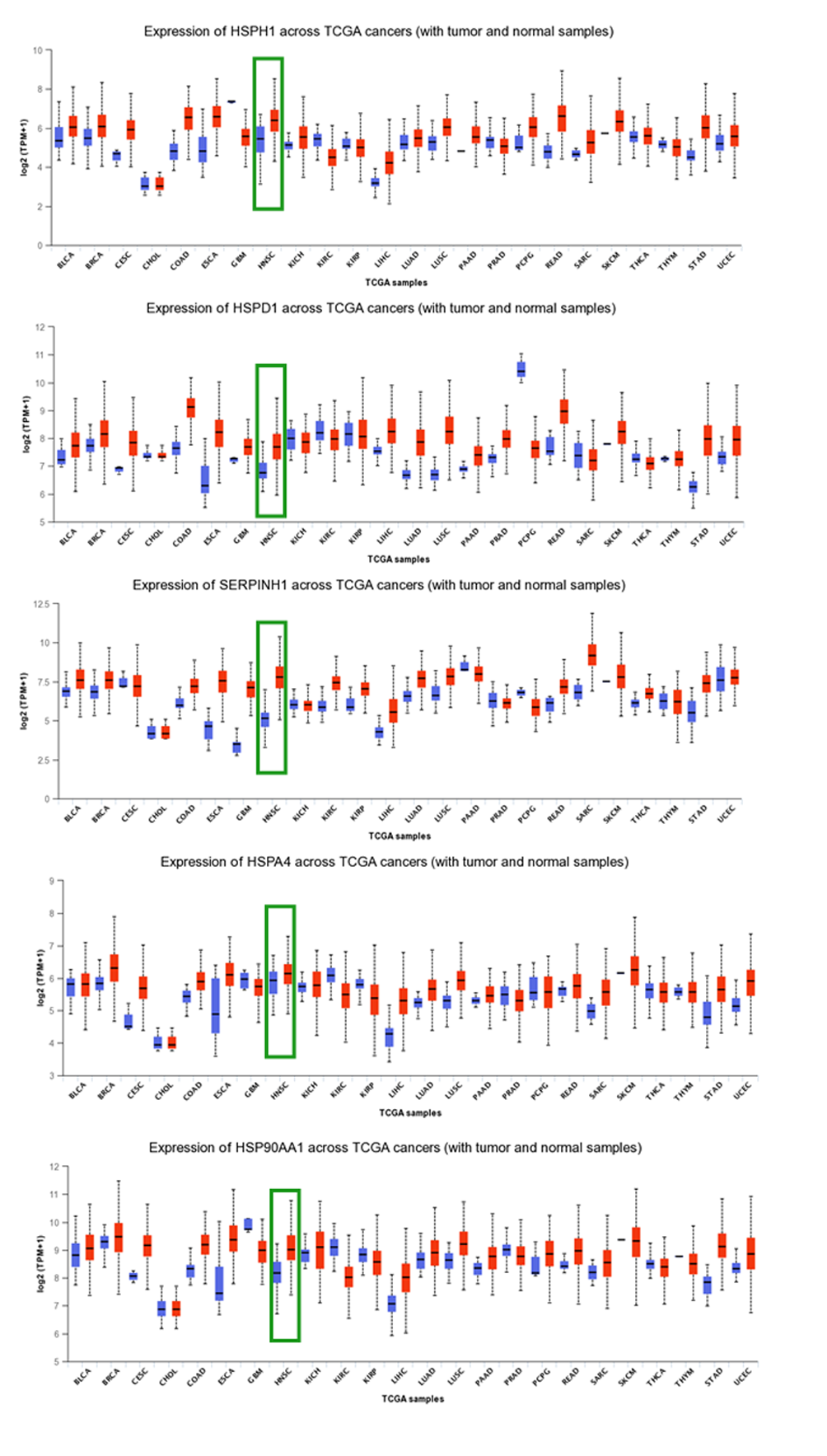

Supplement: Supplementary file 2 — Additional file 2: Fig. S2. Expressions of HSPs across TCGA cancers (with tumor and normal samples). The expression levels of HSPH1, HSPD1, Serpinh1, HSPA4 and HSP90AA1 in pan-cancer. [file 12935_2020_1296_MOESM2_ESM.tif]

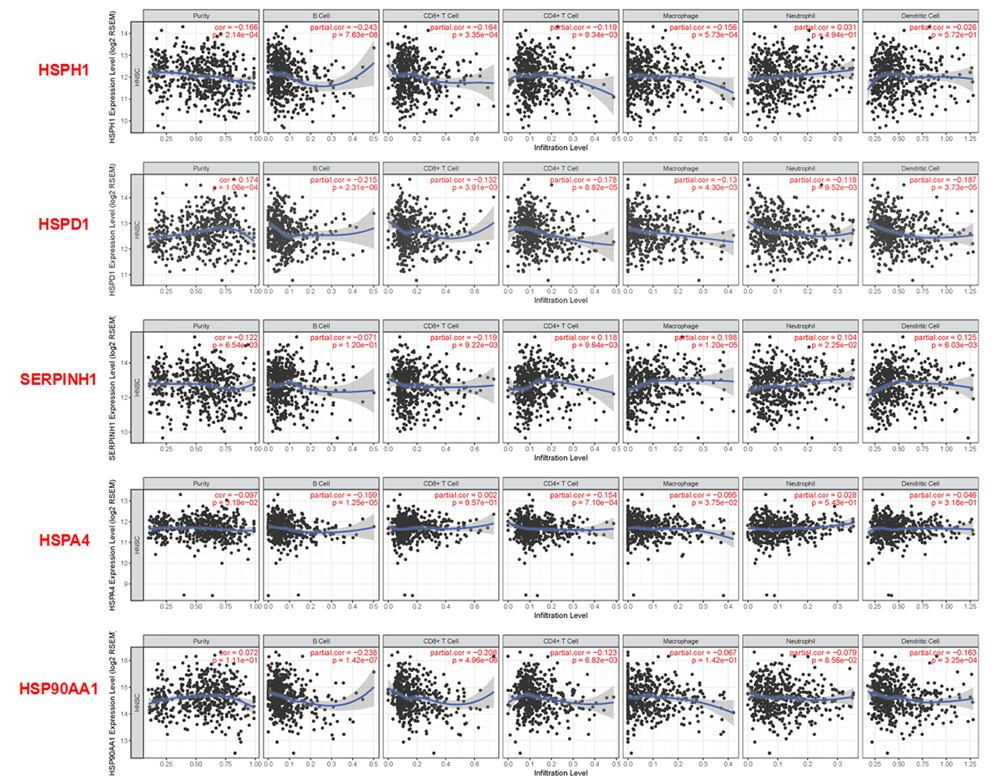

Supplement: Supplementary file 3 — Additional file 3: Fig. S3. The relationships between the HSP expression levels and the levels of immune infiltration in HNSC. [file 12935_2020_1296_MOESM3_ESM.tif]

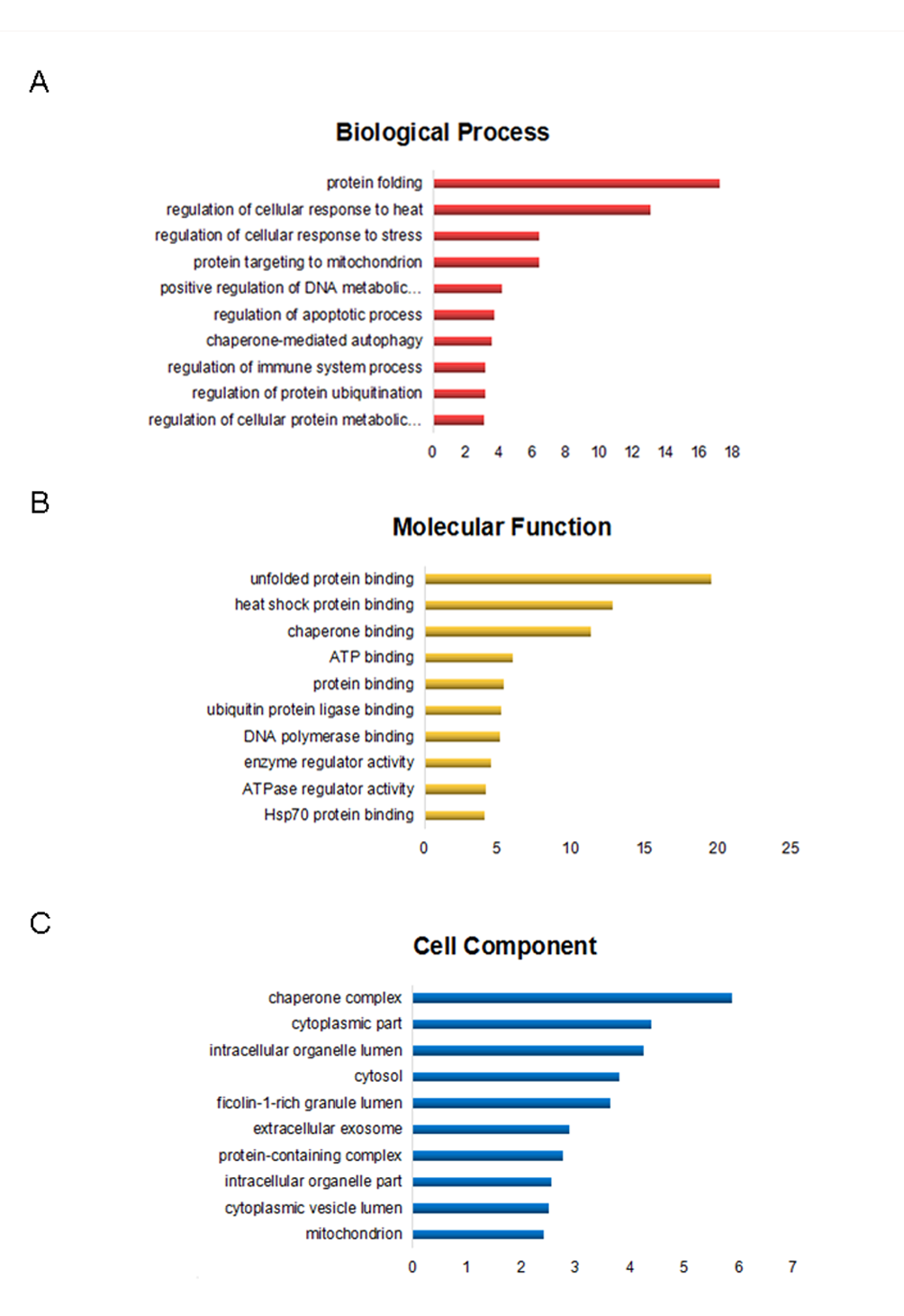

Supplement: Supplementary file 4 — Additional file 4: Fig. S4. Functional Enrichment Analysis of HSPs in patients with HNSC. GO enrichment analysis predicted the function of target genes from three aspects: biological processes (A), cellular components (B), and molecular functions (C). [file 12935_2020_1296_MOESM4_ESM.tif]

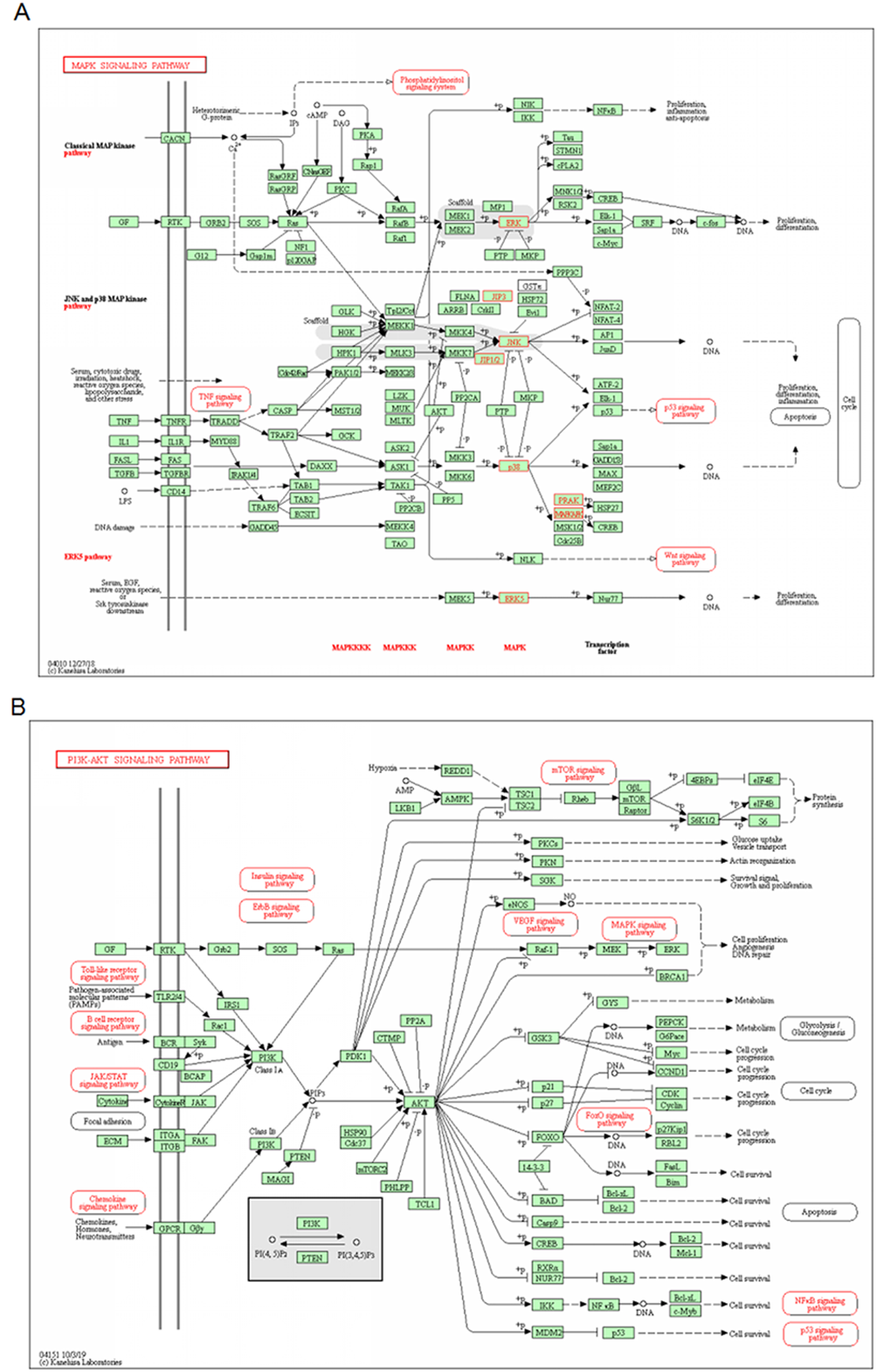

Supplement: Supplementary file 5 — Additional file 5: Fig. S5. p53 signal pathway and cell cycle pathway regulated by HSPs in HNSC. The MAPK signal pathway (A) and PI3K-Akt signaling pathway (B) regulated by HSPs in HNSC are shown. [file 12935_2020_1296_MOESM5_ESM.tif]
